# Glucagon-like peptide 1 and Glucagon-like peptide 2 in relation to osteoporosis in non-diabetic postmenopausal women

**DOI:** 10.1038/s41598-019-50117-z

**Published:** 2019-09-20

**Authors:** María Cristina Montes Castillo, María José Martínez Ramírez, Rubén Soriano Arroyo, Isabel Prieto Gomez, Ana Belén Segarra Robles, Macarena Garrido-Martínez, Piedad Santiago-Fernández, Miguel Delgado Rodríguez

**Affiliations:** 1 Endocrinology and Nutrition, Jaen University Hospital, Av. Ejército Español, sn, Jaén, Spain; 2 Department of Health Sciences, University of Jaen, Campus “Las Lagunillas”, Building B3, Jaén, Spain; 3 Area of Physiology, University of Jaen, Campus “Las Lagunillas”, Building B3, Jaén, Spain; 4 University of Granada, School of Dentistry, Campus “La Cartuja”, Granada, Spain; 5 Department of Preventive Medicine and Public Health, University of Jaen, Campus “Las Lagunillas”, Building B3, Jaén, Spain; 6 CIBERESP, Carlos III Health Institute, Madrid, Spain; 7 Present Address: Endocrinology and Nutrition, La Paz University Hospital, Madrid, Spain; 8 Present Address: Emergency Department, La Paz University Hospital, Madrid, Spain

**Keywords:** Osteoporosis, Gastrointestinal hormones

## Abstract

Osteoporosis results from an imbalance in bone remodeling, which is known to follow a circadian rhythm determined by a functional relationship between intestine and bone tissue. Specific intestinal peptides have been identified as mediators. Glucagon-like peptide 1 and glucagon-like peptide 2, have been associated with bone health. Our main objective was to determine whether postprandial plasma levels of glucagon-like peptide 1, glucagon-like peptide 2 and dipeptidyl-peptidase 4 activity, are associated with osteoporosis in non-diabetic postmenopausal women. We studied non-diabetic postmenopausal women with osteoporosis diagnosed by dual-energy X-ray absorptiometry (cases, n = 43) and age-matched (±1 yr) controls without osteoporosis or a history of osteoporotic fracture (n = 43). We measured postprandial plasma levels of glucagon-like peptide 1, glucagon-like peptide 2, and dipeptidyl-peptidase 4 activity, bone mineral density, and baseline levels of bone remodeling markers and analyzed the food intake using a food-frequency questionnaire. Postprandial glucagon-like peptide 1 values were lower (p < 0.001) in cases, μ (SEM) = 116.25 (2.68), than in controls, μ (SEM) = 126.79 (2.68). Glucagon-like peptide 1 was associated with reduced osteoporosis risk in the crude logistic regression analysis [OR (95% CI) = 0.724 (0.53–0.97), p = 0.031] and adjusted analysis [OR = 0.603 (0.38–0.94), p = 0.027]. We found no association of glucagon-like peptide 2, or dipeptidyl-peptidase 4 activity with osteoporosis. Postprandial glucagon-like peptide 1 levels are related to osteoporosis and osteoporosis risk in non-diabetic postmenopausal women. Further studies are required to verify these findings.

## Introduction

Glucagon-like peptide 1 (GLP1) and glucagon-like peptide 2 (GLP2) are intestinal peptides produced in the digestive system that participate in regulating the different stages of digestion. These peptides have attracted increased research interest in recent years, mainly on GLP1 in relation to glucose metabolism and diabetes mellitus^1,2^ but also on their involvement in other intermediary metabolism pathways, including their effects at bone tissue level and their possible relationship with osteoporosis^3,4^.

Osteoporosis is characterized by bone mass reduction and microarchitecture impairment due to an imbalance in bone remodeling (BR) between bone formation and resorption, increasing the risk of fractures^5^. Under normal circumstances, resorption and formation processes are closely matched to avoid net changes in bone mass^5^.

BR follows a circadian rhythm, with BR markers increasing at night and decreasing during the day, most strongly influencing affecting the resorption mechanism^4^. No relationship has been found between this circadian variation in bone remodeling and the secretion of cortisol, parathormone^6^, or melatonin^7^. It has been proposed that the BR circadian rhythm is influenced by food intake variations. Thus, the rhythm of remodeling is affected by food intake and increases during nocturnal fasting, which mainly affects bone resorption^8,9^ rather than bone formation^10^, and bone resorption was found to be reduced by day-time food intake and increased by nocturnal fasting^11^ independently of age, sex, or menopausal status^12^. It has also been observed that the bone resorption response to glucose is much greater when administered orally *versus* intravenously^13^. Taken together, these data indicate a functional relationship between intestine and bone metabolism that may possibly be mediated by hormones responding to nutrient absorption^14,15^.

Intestinal peptides have been described as key effectors of the acute response of bone metabolism to food consumption^9^. Preliminary data suggest that various intestinal peptides exert positive effects on bone resorption in response to food intake^16^.

A few minutes after food intake, GLP1 and GLP2 are segregated by endocrine L cells distributed throughout the intestinal tract, mainly in the ilium, and reach elevated levels from 30 min after intake^13^. GLP1 is part of the incretin system, which mainly comprises intestinal peptides associated with increased insulin secretion in response to food intake^1^. Other molecules of interest include receptor analogs similar to glucagon-like peptide (GLP1-RA), and dipeptidyl peptidase 4 (DPP4), the enzyme responsible for their metabolism^17,1^. GLP2, which has no incretin effect, acts in the intestine to stimulate mucosal trophism and favor nutrient absorption^3^, and its potential involvement in bone tissue is under investigation^16^.

The action of GLP1 on bone tissue has mainly been investigated in experimental studies. Administration of GLP1 and its receptor-stimulating analog, exendin, was found to reverse bone mass loss in rats^18^, and a later study in rodents observed that exendin favors bone formation and reduces bone resorption^19^. More recently, Meng *et al*. showed that peptide receptor similar to glucagon-1 (GLP1-R) activation improves osteoporosis and promotes osteogenic differentiation into bone marrow stromal cells in an animal model of osteoporosis (tail-suspended rats)^20^.

Studies in diabetic patients have demonstrated that various incretin-effect drugs used in diabetes mellitus may affect bone health. It has also been reported that both GLP1-RA^21,22^ and DPP4 inhibitors^23^ may affect the risk of fracture, although findings have been inconclusive. In addition, a recent meta-analysis associated the administration of liraglutide or lixisenatide with a decreased risk of bone fracture in patients with type 2 diabetes mellitus^24^.

The relationship of GLP2 with bone health has been studied in humans, finding that the intake of mixed food causes a reduction in bone resorption and an associated increase in GLP2 and that GLP2 treatment significantly reduces bone resorption^8,15^ and improves bone mass^10^.

Although a relationship has been demonstrated between DPP4 and osteoporosis in non-diabetic postmenopausal women^25,26^ there is little evidence on the association of GLP1 with osteoporosis in humans with no glucose metabolism disorder. Confirmation of this relationship would be of interest, especially in relation to GLP1, because drugs based on these peptides are used in diabetes mellitus and may be of potential value in the treatment of osteoporosis. Therefore, the main objective of this study was to determine whether GLP1 and also GLP2 and the enzyme responsible for metabolism, DPP4, are related to the presence of osteoporosis diagnosed according to bone mass criteria in non-diabetic postmenopausal women.

## Methods

### Study design

We conducted a case-control study with non-diabetic postmenopausal women with and without osteoporosis, matched 1:1 by age (±1 yr.).

We estimated a sample size of 38 patients per group based on the next statistical assumptions: needed to detect a significant difference (alfa error of 5%) between two means (106.3 vs. 92.2) with a common standard deviation of 12 based on the paper by Wojcik *et al*.^27^ and a statistical power of 90%. Finally, we enrolled 86 women: 43 cases and 43 controls. Cases were women diagnosed with osteoporosis and controls were women without osteoporosis or a history of fracture.

We recruited volunteers from among patients who attended outpatient clinics of different specialties at our hospital between January 2015 to January 2016 and who met study eligibility criteria (see below).

Inclusion criteria for cases were: (1) female with age <70 yrs, (2) diagnosis by bone mass measurement with dual energy X-ray absorptiometry (DEXA) of osteoporosis, defined by bone mineral density (BMD) T score value ≤−2.5 standard deviations measured at femoral or lumbar sites; (3) absence of diabetes mellitus or pre-diabetes status (based on glycosylated hemoglobin and baseline fasting glycemia according to the criteria of the American Diabetes Association)^28^; and (4) postmenopausal status, defined by the presence of amenorrhea for more than one year.

Exclusion criteria were: (1) diagnosis of secondary osteoporosis^29^; (2) presence of any endocrinal disease and/or food behavior disorder; (3) pregnancy; (4) hospitalization during the previous six months; (5) diagnosis of severe cancer; (6) diagnosis of ileocolic disease (inflammatory bowel disease, intestinal malabsorption, or intestinal resection or fistulae); (7) diagnosis of stage IV chronic kidney disease: Modification of diet in renal disease-4 (MDRD-4) measured glomerular filtration rate (GFR) <30 mL/min/1.73 m^2 ^^30^; (8) active treatment with: biological factors; anti-diabetic drug, including DDP4 inhibitors, or GLP-1 or GLP-2 analogs; cholestyramine; anticonvulsants; rifampicin; antacids; antineoplastic; corticoids; or anti-osteoporotic drugs.

Controls were age-matched (±1 yr.) non-diabetic postmenopausal women with DEXA-confirmed absence of osteoporosis and no history of low-energy fracture. Other exclusion criteria were the same as for cases.

We gathered data on: the participants’ history of disease and drug consumption; their dietary intake, using a semi-quantitative food frequency questionnaire adapted to the Spanish population^31^; and their weight (kg), height (cm), and body mass index (BMI) (Kg/m^2^).

### Bone mass determination

All participants underwent densitometry with LUNAR DPX GE HC densitometer in lumbar spine (vertebras L1-L4) and left femoral neck. We determined the T-scores and Z-scores. considering osteoporosis as a function of T-score when BMD values were ≤−2.5 standard deviations (T-score ≤ −2.5) measured at one or both sites.

### Analytical determinations

A blood sample was drawn from the antecubital vein at baseline after >8 h fasting for the measurement of bone metabolism and general biochemistry parameters^32^. A second sample was drawn on the same day at 30 min after^13,33^ the intake by participants of the same complete and chemically defined nutritional preparation of carbohydrates, proteins, and lipids (Resource HP/HC, NESTLE HEALTH SCIENCE) (see supplementary information) for peptide and DPP4 activity determinations, because intestinal peptide levels are very low under fasting conditions.

#### Bone metabolism determination and general biochemistry parameters

The following metabolism and BR parameters were analyzed in the hospital laboratory after >8 h fasting^32^: plasma calcium and phosphorus (mg/dL), 25-OH vitamin D (ng/mL), intact parathyroid hormone (PTHi) (pg/mL), osteocalcin (ng/mL), procollagen type I aminoterminal propeptide (PINP) (ng/mL), and type I collagen C-terminal telopeptide (CTX) (ng/mL), and usual biochemical values, including glycosylated hemoglobin (%), basal glucose (mg/dL), and serum albumin (g/dL).

#### GLP1 and GLP2 determination

GLP1 (pg/mL) and GLP2 (pg/mL) were measured in the physiology laboratory of the University of Jaen (Spain). Immediately before postprandial blood samples were drawn, 10 μL/mL of a DPP4 inhibitor (DPP4/DPP4-010, Linco Research Inc, St Charles, Missouri, USA) were added to the tubes, following Hattori *et al*.^34^. Plasma samples were then obtained by placing the tubes in ice and immediately centrifuging them at 4 °C and 3,000 × g for 30 min followed by their storage at −80 °C. Specific BIONOVA® commercial kits were used to determine total (cleaved and uncleaved) GLP-1 and GLP-2 levels using ELISA techniques.

#### DPP4 activity determination

DPP4 activity was determined at the above physiology laboratory in blood samples drawn at 30 min after consumption of the aforementioned nutritional preparation into tubes with no DPP4 inhibitor, using a fluorimetry assay (Sigma-Aldrich DPP4 Activity Assay Kit) based on hydrolysis by the enzyme of the H-Gly-Pro-4-methoxy-β-naphthylamide substrate, which releases β-naphthylamide, measuring its fluorescence at 345 nm excitation and 412 nm emission wavelengths after incubation at 37 °C. Values were expressed as pmol of β-naphthylamine released per minute of incubation and per mL of plasma.

### Statistical analysis

The Student’s t-test was used to compare means between cases and controls. Linear regression analysis was performed to evaluate the prediction by bone remodeling parameters of peptide levels separately in cases and controls. Conditional logistic regression analysis was used to assess associations between the different peptides and osteoporosis, adjusting for potential confounders. Program Stata 14 SE (College Station, TX, US) was used for data analyses.

### Ethics statement

All enrolled patients signed informed consent to participation in the study, which was approved by the Research Ethics Committee and followed all recommendations of the Helsinki Convention.

The study was approved by local ethical committee “Comité de Ética de la Investigación de Jaén”, (date: 10-30-2014).

### Ethical approval

All procedures performed in studies involving human participants were in accordance with the ethical standards of the institutional research committee and with the 1964 Helsinki declaration and its later amendments or comparable ethical standards.

This article does not contain any studies with animals performed by any of the authors.

### Informed consent

Informed consent was obtained from all individual participants included in the study.

## Results

Table 1 lists results of the descriptive analysis. The mean age (SEM) was 58.74 (0.63) yrs for cases and 58.76 (0.63) yrs for controls. No statistically significant difference was found between cases and controls in mean age, history of disease or pharmacological treatment (data not shown in table), glycosylated hemoglobin, alcohol, tobacco consumption, or dietary intake (energy and macronutrients). Weight and BMI values were significantly lower (*p* < 0.001) in cases than in controls.

**Table 1:** Descriptive analysis.

Variables	Controls (*n* = *43)*	Cases (*n* = *43)*	*p value*
Age (yrs), mean (SEM)	58. 8 (0.6)	58.7 (0.6)	0.98
**Work situation, n (%)**			
Active	21 (48.8)	29 (67.4)	0.168
Retired or unemployed	5 (11.6)	5 (11.6)	
Housewife	17 (39.5)	9 (20.9)	
**Tobacco n (%)**			
No	35 (81.4)	33 (76.3)	0.702
<1–4 cigarettes/day	0 (0.0)	2 (4.7)	
5–10 cigarettes/day	3 (7.0)	3 (7.0)	
>10 cigarettes/day	4 (9.3)	4 (9.3)	
Not known/No response	1 (2.3)	1 (2.3)	
**Alcohol**			
No	37 (86.0)	32 (74.4)	0.152
Occasional	2 (4.7)	8 (18.6)	
<10 g day	3 (7.0)	3 (7.0)	
Energy (Kcal/d), mean (SEM)	2723 (217.90)	2604 (166.60)	0.665
Carbohydrates (g/d), mean (SEM)	295.5 (30.86)	263.3 (20.16)	0.387
Proteins (g/day), mean (SEM)	123.5 (8.89)	123.3 (11.0)	0.99
Total fat (g/day), mean (SEM)	114.1 (9.84)	111.5 (6.61)	0.832
Weight (Kg), mean (SEM)	75.06 (2.13)	63.39 (1.84)	<0.001
Height (cm), mean (SEM)	157.6 (0.84)	156.19 (1.21)	0.336
BMI (Kg/m^2^), mean (SEM)	30.29 (0.90)	26.04 (0.74)	<0.001
Glycosylated hemoglobin, mean (SEM)	5.53 (0.05)	5.51 (0.04)	0.85

BMI: body mass index.

Table 2 compares means values of blood variables between cases and controls, showing significantly lower (p < 0.001) GLP1 levels in cases (μ [SEM]) = 116.75 [2.68]) than in controls (μ [SEM] = 126.79 ± 2.68). No statistically significant between-group differences were found in plasma GLP2, DPP4, 25-OH vitamin D, PTHi, calcium (albumin-corrected), or phosphorus levels. As expected, cases and controls significantly differed (*p* < 0.001) in densitometry and BR parameters (osteocalcin, PINP, and CTX).

**Table 2:** Comparison of means of main variables between cases and controls.

Variable	Controls (n = 43)	Cases (n = 43)	*p value*
Intestinal peptides	Mean (SEM)	Mean (SEM)	
GLP1 (pg/mL)	126.79 (2.68)	116.75 (2.68)	<0.001
GLP2 (pg/mL)	308.91 (13.27)	310.49 (14.3)	0.933
DDP4 (pmol/min/mL)	7643.02 (223.2)	7711 (207.9)	0.822
**Bone remodeling markers**			
Albumin-corrected serum Ca (mg/dL)	9.26 (0.05)	9.31 (0.03)	0.373
Serum phosphorus (mg/dL)	3.24 (0.05)	3.36 (0.08)	0.234
25OHD (ng/mL)	18.63 (1.05)	19.3 (30)	0.652
PTHi (pg/mL)	58.51 (7.87)	47.88 (2.75)	0.206
Osteocalcin (ng/mL)	19.36 (1.07)	23.23 (1.11)	0.014
PINP (ng/mL)	39.04 (2.16)	51.24 (3.05)	0.001
CTX (ng/mL)	0.322 (0.02)	0.481 (0.03)	<0.001
**Bone mass**			
Lumbar spine t-score	0.185 (0.10)	−2.823 (0.10)	<0.001
Lumbar spine z-score	1.274 (0.11)	−1.344 (0.14)	<0.001
Femur t-score	0.302 (0.12)	−1.92 (0.13)	<0.001
Femur z-score	0.89 (0.11)	−1.11 (0.13)	<0.001

GLP1: Glucagon-like 1.

GLP2: Glucagon-like 2.

DPP4: dipeptidyl peptidase 4 activity.

25OHD: 25-OH vitamin D.

CTX: Type I collagen C-terminal telopeptide.

PINP: Type I collagen I N-terminal propeptide.

BMI: body mass index.

Table 3 exhibits results of the linear regression analysis for GLP1, GLP2, and DPP4 levels and BR markers. We found a positive correlation between GLP1 and CTX that was significant in cases (*p* = 0.011) and close to significant in controls (p = 0.054), and a significant negative correlation between GLP1 and PINP in controls (*p* = 0.043). GLP2 was also positively correlated with osteocalcin in controls (*p* = 0.04). DPP4 was positively correlated with PINP (*p* < 0.001) and CTX (*p* = 0.022) in controls.

**Table 3:** Association of GLP1, GLP2, DPP4 (postprandial levels) with bone remodeling parameters (fasting levels) in cases and in controls.

	Controls (n = 43)		Cases (n = 43)	
	β coefficient (SE)	*p value*	β Coefficient (SE)	*p value*
**Dependent variable: GLP1 (pg/mL)**				
Osteocalcin (ng/mL)	−0.123 (0.55)	0.82	0.070 (0.59)	0.905
PINP (ng/mL)	−0.699 (0.33)	0.043	−0.365 (0.21)	0.098
CTX (ng/mL)	58.59 (29.49)	0.054	49.27 (18.33)	0.011
**Dependent variable: GLP2 (pg/mL)**				
Osteocalcin (ng/mL)	5.74 (2.69)	0.04	−2.53 (3.51)	0.476
PINP (ng/mL)	−3.03 (2.69)	0.07	1.42 (1.29)	0.277
CTX (ng/mL)	−80.30 (143.67)	0.579	−89.95 (109.9)	0.621
**Dependent variable DPP4 (pg/mL)**				
Osteocalcin (ng/mL)	−69.93 (41.9)	0.104	−15.16 (47.55)	0.752
PINP (ng/mL)	99.19 (25.3)	<0.001	15.21 (17.46)	0.389
CTX (ng/mL)	−534.2 (2237.6)	0.022	1689.03 (1486.4)	0.263

*SE (Standard Error)*.

Linear regression analysis.

Table 4 displays results of the crude and adjusted conditional logistic regression analyses on the relationship with the presence of osteoporosis of intestinal peptides and DPP4, BR parameters, dietary intake, and BMI. GLP1 was associated with a significant reduction in osteoporosis risk after adjustment for BMI and CTX as confounders (OR [95% CI] = 0.724 [0.53–0.97]).

**Table 4:** Relationship of main variables with osteoporosis.

Variable	Crude analysis		Adjusted analysis^a^	
	OR (95% CI)	*p value*	OR (95% CI)	*p value*
GLP1 (per 10) (pg/mL)	0.724 (0.53–0.97)	0.031	0.603 (0.38–0.94)	0.027
GLP2 (per 100) (pg/mL)	1.03 (0.55–1.96)	0.912	0.99 (0.30–3.23)	0.988
DPP4 (per 1000) **(**pmol/min/ml)	1.06 (0.71–1.57)	0.766	0.89 (0.42–1.90)	0.775
Albumin-corrected calcium (mg/dL)	2.03 (0.42–9.89)	0.380	3.47 (0.20–59.1)	0.389
25OHD (ng/mL)	1.01 (0.03)	0.635	1.01 (0.9–1.13)	0.807
Osteocalcin (ng/mL)	1.1 (1.02–1.19)	0.017	1.05 (0.90–1.23)	0.505
PINP (ng/mL)	1.05 (1.01–1.08)	0.005	1.02 (0.96–1.07)	0.486
CTX (ng/mL)	562.4 (12.93–24461	0.001	297.98 (4.32–20531)	0.008
Energy intake (Kcal/day)	0.99 (0.99–1.00)	0.718	0.99 (0.99–1.00)	0.723
Protein intake (g/day)	1.00 (0.99–1.00)	0.949	1.00 (0.99–1.01)	0.618
BMI (Kg/m^2^)	0.73 (0.59–0.88)	0.002	0.75 (0.61–0.91)	0.005

^a^Adjusted for BMI and CTX.

Logistic regression analysis.

## Discussion

The main finding of this study was the association between plasma GLP1 levels and osteoporosis in non-diabetic postmenopausal women. Postprandial GLP1 values were significantly lower in non-diabetic postmenopausal women with than without osteoporosis, and higher values were significantly associated with a reduction in osteoporosis risk in the crude and adjusted logistic regression analyses. The presence or risk of osteoporosis was not associated with GLP2 levels or DPP4 activity.

The existence of a relationship between bone tissue and intestinal peptides was also supported by the following findings: (1) a positive correlation of GLP1 with the BR marker CTX that was significant in cases and close to significant in controls, and a negative correlation of GLP1 with PINP in controls; (2) a positive and significant association of GLP2 with osteocalcin in controls; and (3) a positive association of DPP4 activity with PINP and its negative association with CTX in controls.

The main study limitation is that we performed only one analytical determination of peptides and other bone metabolism markers. Besides the results obtained for GLP1, a strength of this study is that it appears to be the first to compare postprandial levels of GLP1 and GLP2 peptides between non-diabetic postmenopausal women with and without osteoporosis.

Our results for GLP1 are conclusive, observing a clear association with osteoporosis in the comparison of means and in the conditional logistic regression. The association between GLP1 and bone tissue was previously evidenced in preclinical studies.

GLP-1 must bind with its receptor to exert its metabolic effects^14^ and is rapidly inactivated by enzyme DPP4, resulting in inactive GLP1_9–36_ with low affinity for GLP1-R^35^. GLP1-R has been detected in pancreatic islets, lung, stomach, kidney, hypothalamus, and heart but not in liver, adipose tissue, or skeletal muscle^35^, although Nuche-Berenguer *et al*. reported that GLP1 can act directly on cultured osteoblasts (MC3T3-E1 osteoblastic cells) *via* a membrane receptor^36^. In addition, Pacheco-Pantoja *et al*. (2011) observed the expression of GLP1-R in different human osteoblast cell lines and demonstrated their influence on the secretion of osteocalcin, alkaline phosphatase, and PINP^37^.

The molecular mechanisms underlying the effects of GLP1 on bone tissue have not yet been elucidated. GLP1 or GLP1-RA may act directly on bone *via* functional GLP1-R expressed by bone cells^36,37^ or indirectly through an increased production of calcitonin by thyroid C cells, inhibiting bone resorption^38^.

The action of GLP1 and GLP1-RA on bone tissue has been investigated in animal and *in vitro* studies. GLP1-R knockout mice showed densitometry-measured osteopenia and bone fragility and increased bone histomorphometry-evaluated resorption and osteoclastic activity^39^. In another study^40^, GLP1-R *knockout* mice evidenced significantly reduced bone strength, rigidity, and quality in comparison to *wild-type* mice, with a less mature collagen matrix and inferior intrinsic bone properties, although no statistically significant difference in bone mineral quantity was observed; the authors described GLP1-R as likely responsible for bone tissue resistance and quality^40^.

In 2011, Nuche-Berenguer *et al*. reported that the administration of GLP1 and exendin (receptor analog) improved lipid and glucose metabolism and increased the expression of genes encoding osteocalcin and osteoprotegerin, reversing bone mass loss^18^. Ma *et al*. observed that exendin administration in ovariectomized rats exerted a protective effect against osteoporosis, modulating the balance between bone resorption and formation^19^. More recently, exendin was found to have an anabolic effect on bone tissue, suggesting that GLP1-R participates in bone marrow stromal cell differentiation into osteoblasts^20^.

Our study confirms that GLP1 is related to osteoporosis in non-diabetic postmenopausal women, finding a lower release of GLP1 in response to food in cases than in controls and observing that GLP1 was associated with a significant reduction of around 27% in osteoporosis risk.

However, our findings on the relationship between GLP1 and BR markers were unexpected. We found that GLP1 was positively associated in cases with CTX, a bone resorption parameter, and was negatively associated in controls with PINP, a bone formation parameter. A recent study of overweight/obese men reported that GLP1 and gastric inhibitory polypeptide (GIP) reduced their CTX levels and that the co-infusion of both peptides had a synergistic effect on their CTX levels and bone resorption^41^. In another investigation, obese women who had lost weight after following a hypocaloric diet were four-fold less likely to lose bone mass if treated with liraglutide, showing an increase in PINP but no change in CTX in comparison to the women not treated with this GLP1 analog^42^.

The discrepancy between these results may have various explanations: (1) our main objective was to associate GLP1 with osteoporosis, not directly with BR markers; (2) postmenopausal osteoporosis is a bone disorder with intense BR^43^, increasing all BR markers, with a final predominance of bone resorption; and (3) we studied postprandial levels of peptides but fasting values of BR parameters, and GLP1 secretion likely changes in response to food intake. Furthermore, our results are in at least partial agreement with the study of Pacheco-Pantoja in osteoblast cell lines, which reported a reduction in PINP secretion after stimulation with GLP1^37^. In addition, a polymorphism in GLP1-R has been found to influence osteoporosis risk^44^ and may cause a dissociated response of bone tissue to GLP1 and its analogs. A meta-analysis in 2013 reported that different GLP1 analogs had opposite effects on the risk of osteoporotic fracture^45^, and a more recent meta-analysis concluded that only two GLP1 analogs, liraglutide and lixisenatide, reduced bone fracture risk and that their effect depended on the treatment duration^24^.

As in the case of GLP1, GLP2 has been shown by various researchers to exert beneficial effects on bone tissue^46^. However, we found no association between GLP2 and osteoporosis, although we did observe a positive and significant association between GLP2 and osteocalcin in our control group. GLP1 and GLP2 are secreted in a 1:1 ratio by intestinal endocrine L cells, and a similar between-group difference in GLP-2 might therefore be expected^33^. Fasting plasma levels of the active forms of these peptides are 5–10 pM for GLP1 and 15–20 pM for GLP2, and these values can be 2- to 5-fold higher after intake, with GLP2 being more stable than GLP1^33^. These baseline differences may explain our finding of disparities in the levels of these peptides. Other studies have described a similar divergence in their values^47,48^.

The effects of GLP2 on bone tissue have been widely studied in humans. The underlying molecular mechanisms of its action have yet to be elucidated^4^, but the presence of peptide receptor similar to glucagon-2 has been proposed in some osteoblast cell lines that showed increased osteocalcin synthesis in response to GLP2^37^. In studies by Henriksen *et al*., the intake of a mixture of nutrients by healthy volunteers reduced bone resorption and produced the parallel secretion of GLP1 and GLP2, and the intravenous injection of different doses of GLP2 in 60 postmenopausal women reduced bone resorption but had no effect on bone formation parameters^15^. In a later study by the same group, the administration of GLP2 for 14 days to healthy postmenopausal women was found to be a safe treatment that significantly reduced bone resorption and did not affect bone formation, with osteocalcin levels remaining stable^8^. A trial in which 160 postmenopausal women were treated with GLP2 described an increase in hip bone density and reduction in nocturnal CTX concentrations at day 120 post-injection, with no modification in osteocalcin^49^.

In contrast, we found a significant relationship of osteoporosis with GLP1 but not with GLP2, which may be attributable to the difference in their actions. The principal actions of GLP2 are at intestinal level (trophic effect and stimulation of intestinal absorption), and its receptors are mainly present at intestinal and brain level^50,51^, although their presence is suspected, with no clear evidence, in human bone tissue cells^46^. For its part, GLP1 exerts its actions at pancreatic level, mainly with incretin effect, and its receptors are present in a larger number of tissues^33^ and in osteoblasts^37^, with direct effects on bone cells^46^.

Our observation that GLP2 was positively associated with osteocalcin in the women without osteoporosis is consistent with the increase in osteocalcin previously observed in post-menopausal women after the subcutaneous administration of GLP2 under fasting conditions^52^ and also with the aforementioned report on increased osteocalcin in osteoblast cell lines after GLP2 treatment^37^.

In the present study, DPP4 activity did not differ between participants with *versus* without osteoporosis, while it was positively associated with PINP and negatively associated with CTX but only among those without osteoporosis. Kim *et al*. recently observed an association between DPP4 activity levels and osteoporotic fracture risk in non-diabetic postmenopausal women, finding that high levels were associated with elevated BR markers; the authors indicated that bone formation and resorption was influenced by certain DPP4 substrates^25^. Zheng *et al*. studied 744 postmenopausal women with no glucose metabolism disorder and found higher DPP4 activity in patients with osteoporosis and a positive association with osteocalcin and CTX. The discrepancy between these results may be attributable to differences in their sample sizes or to the determination of DPP4 activity after fasting by Zheng^26^.

In conclusion, postprandial GLP1 levels are significantly reduced in non-diabetic postmenopausal women with osteoporosis, and higher postprandial GLP1 levels are associated with reduced osteoporosis risk in this population. This study contributes new data on the relationship between osteoporosis and intestinal peptides in humans and verifies the association between GLP1 and osteoporosis in non-diabetic postmenopausal women. These results suggest that GLP1 analog molecules, currently prescribed for diabetes mellitus, may potentially represent an alternative therapeutic approach to osteoporosis. No association was observed between osteoporosis and GLP2 levels or DPP4 activity, and the relationship of GLP1, GLP2, and DPP4 with bone remodeling markers remains unclear. Further research is warranted on the links between intestinal peptides and bone tissue.

## Supplementary information

Supplementary material
