# Supplementary material for: Glucagon-like peptide 1 and Glucagon-like peptide 2 in relation to osteoporosis in non-diabetic postmenopausal women
Source: Sci Rep. 2019 Sep 20;9:13651. doi: 10.1038/s41598-019-50117-z (PMC6754449; doi:10.1038/s41598-019-50117-z)
Supplement: Supplementary file 1 — Supplementary material [file 41598_2019_50117_MOESM1_ESM.pdf]

“Glucagon-like peptide 1 and Glucagon-like peptide 2 in relation to osteoporosis in non-diabetic postmenopausal women”

María Cristina Montes Castillo. cristinamontescastillo86@gmail.com https://orcid.org/0000-0001-6375-5433

María José Martínez Ramírez. mjmartin@ujaen.es https://orcid.org/0000-0001-6767-3881

Rubén Soriano Arroyo. soriano.granada@gmail.com https://orcid.org/0000-0002-2910-1804

Isabel Prieto Gomez. iprieto@ujaen.es https://orcid.org/0000-0003-0624-9504

Ana Belén Segarra Robles. absegarra@ujaen.es https://orcid.org/0000-0002-9409-6052

Macarena Garrido-Martínez. maky_109@hotmail.com https://orcid.org/0000-0002-3190-8404

Piedad Santiago-Fernández. psantiagofernandez63@gmail.com https://orcid.org/0000-0001-6440-7174

Miguel Delgado Rodríguez. mdelgado@ujaen.es https://orcid.org/0000-0002-3838-2548

Supplementary material

Nutritional composition of Resource HP/HC (NESTLE HEALTH SCIENCE)

1 package of 200 mL contains:

- 1.6 Kilocalories/mL
- 320 kilocalories
- 20 g proteins (P)
- 32 g carbohydrates (CHO)
- 12.4 g lipids (L)
- Dietary fiber: 0 g
- Calorie distribution: P/CHO/L/F: 25/40/35/0
